# Origin of fast ion diffusion in super-ionic conductors

**DOI:** 10.1038/ncomms15893

**Published:** 2017-06-21

**Authors:** Xingfeng He, Yizhou Zhu, Yifei Mo

**Affiliations:** 1 Department of Materials Science and Engineering, University of Maryland, College Park, Maryland 20742, USA; 2 University of Maryland Energy Research Center, University of Maryland, College Park, Maryland 20742, USA

## Abstract

Super-ionic conductor materials have great potential to enable novel technologies in energy storage and conversion. However, it is not yet understood why only a few materials can deliver exceptionally higher ionic conductivity than typical solids or how one can design fast ion conductors following simple principles. Using *ab initio* modelling, here we show that fast diffusion in super-ionic conductors does not occur through isolated ion hopping as is typical in solids, but instead proceeds through concerted migrations of multiple ions with low energy barriers. Furthermore, we elucidate that the low energy barriers of the concerted ionic diffusion are a result of unique mobile ion configurations and strong mobile ion interactions in super-ionic conductors. Our results provide a general framework and universal strategy to design solid materials with fast ionic diffusion.

## Abstract (web summary)

*Ab initio* atomistic modelling reveals that fast ion diffusion in super-ionic conductors is mediated by concerted migration of multiple ions. On the basis of this understanding, a universal strategy for designing fast ion conductors is proposed and demonstrated.

Solid materials with fast ionic transport are indispensable components in electrochemical energy storage and conversion devices such as batteries, fuel cells and electrochemical membranes^1^,^2^,^3^,^4^,^5^,^6^, which are critical in the societal shift to renewable energy. These electrochemical devices can further improve through the use of super-ionic conductor (SIC) materials, which have several orders of magnitude higher ionic conductivity than typical solids. For example, lithium SICs, including Li_10_GeP_2_S_12_ (LGPS)^7^, Li_7_P_3_S_11_ (ref. 8), lithium garnet (for example, Li_7_La_3_Zr_2_O_12_ (refs 9, 10)), and Li^+^-conducting NASICON (for example, Li_1.3_Al_0.3_Ti_1.7_(PO_4_)_3_ (ref. 11)), achieve high Li ionic conductivity, ∼1–10 mS cm^−1^ at room temperature (RT), and low activation energy, ∼0.2–0.3 eV. These SIC materials are promising solid electrolytes for the development of next-generation all-solid-state Li-ion batteries, which provide improved safety, higher energy density, and better thermal stability than current organic electrolyte-based Li-ion batteries^6^,^7^,^12^,^13^. Despite significant research efforts, only a few materials out of tens of thousands of known inorganic materials have been identified as SICs. It is of great scientific interests to understand why these SICs can achieve several orders of magnitude faster ionic diffusion than other solid materials, and to enable a rationally guided materials design strategy for fast ion conductors.

Current understanding of ionic diffusion in solids is based on the classical diffusion model, which describes ionic transport as the hopping of individual ions from one lattice site to another through inter-connected diffusion channels in the crystal structural framework^14^ (Fig. 1). The crystal structural framework determines the energy landscape of the ion migration. During ion diffusion, a mobile ion migrates through the energy landscape, and the highest energy of the energy landscape along the diffusion path determines the energy barrier *E*_a_ of ionic diffusion. A low activation energy *E*_a_ and a high concentration *n*_c_ of mobile ion carriers (such as vacancies or interstitials) are required to achieve high ionic conductivity *σ*, which is proportional to *n*_c_·exp(−*E*_a_/*k*_B_*T*) at temperature *T*. On the basis of this classical diffusion model, current research efforts in the design and discovery of fast ion conductors target materials with crystal structural frameworks that yield an energy landscape of low barriers. For example, the structural framework with body-centred cubic (bcc) anion packing yields the flattest energy landscape with the lowest Li^+^ migration barrier, for example, ∼0.2 eV in lithium-containing sulfides, whereas non-bcc structural frameworks such as in face-centred cubic or hexagonal close-packed exhibit significantly higher energy barriers^13^. Unfortunately, bcc anion packing is a rare structural feature in Li-containing oxides and sulfides, and among known Li SICs is only found in LGPS and Li_7_P_3_S_11_. Other well-known SICs, such as lithium garnet and NASICON, do not exhibit bcc anion packing, but still achieve high Li^+^ ionic conductivity of ∼1 mS cm^−1^ at RT.

Super-ionic conduction is known to be activated at high mobile-ion concentration *n*_c_ and in specific mobile ion sublattice configuration achieved through materials doping. For example, Li garnet achieves the highest RT Li conductivity, *σ*_RT_=∼0.1 to 1 mS cm^−1^ (*E*_a_=∼0.3 eV), at 6.4–7.0 Li per formula unit in the doped, cubic-phase Li_7_La_3_Zr_2_O_12_ compositions^10^,^15^,^16^, whereas Li_5_La_3_Ta_2_O_12_ composition of the same crystal structural framework only exhibits *σ*_RT_=∼10^−3^ mS cm^−1^ (*E*_a_=∼0.5 eV)^17^. Li^+^-conducting NASICON Li_1+*x*_Al_*x*_Ti_2−*x*_(PO_4_)_3_ achieves high ionic conductivity *σ*_RT_=∼1 mS cm^−1^ (*E*_a_=∼0.3 eV) at *x*=0.2–0.3 (ref. 11), whereas LiTi_2_(PO_4_)_3_ composition has only *σ*_RT_=∼10^−3^ mS cm^−1^ (*E*_a_=∼0.45 eV)^11^,^18^. Therefore, the super-ionic conduction in these materials is only activated at certain doped compositions with particular Li^+^ sublattice ordering. However, the classical diffusion model, which predicts similar migration barriers for the same crystal framework, fails to capture such super-ionic conduction in these materials. For example, the classical model cannot explain why Li^+^ migrations suddenly exhibit significantly lower activation energy barriers in the same crystal structural framework with similar energy landscape as seen in doped Li garnet and NASICON. The answer to this question may help guide the design of SIC materials, especially those with distinctive crystal structural frameworks that deviate from the optimal bcc anion packing.

In this study, we reveal the origin of fast ionic diffusion in SIC materials with distinctive structural frameworks. We demonstrate a general understanding of fast ionic diffusion across a range of materials using a diffusion model with explicit consideration of the unique mobile-ion sublattice at super-ionic states. Furthermore, we establish a simple conceptual framework for activating fast ion conduction with low migration barriers through materials design, which is generally applicable to any ion-conducting materials.

## Results

### Concerted ion migration in super-ionic conductors

**Figure 2:**
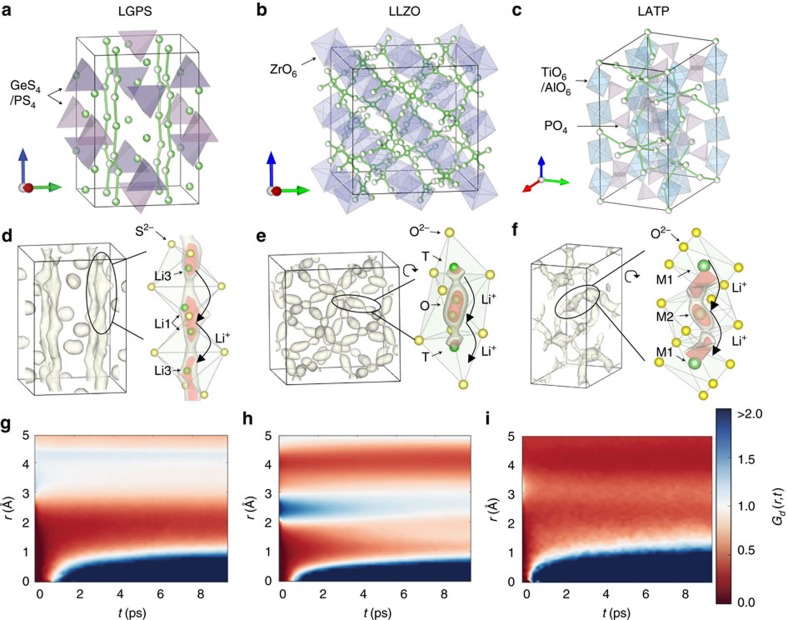
Li ion diffusion in super-ionic conductors. (**a**–**c**) Crystal structures of (**a**) LGPS, (**b**) LLZO and (**c**) LATP marked with Li sites (partially filled green spheres), Li^+^ diffusion channels (green bars), and polyanion groups (purple and blue polyhedra). (**d**–**f**) The probability density of Li^+^ spatial occupancy during AIMD simulations. The zoom-in subsets show the elongation feature of probability density along the migration channel (Li: green; O/S: yellow). The isosurfaces are 6*ρ*_0_, 6*ρ*_0_, 2*ρ*_0_ for LGPS, LLZO, LATP, respectively, where *ρ*_0_ is the mean probability density in each structure and the inner isosurfaces have twice the density of the outer isosurfaces. (**g**–**i**) Van Hove correlation functions of Li^+^ dynamics on distinctive Li ions during AIMD simulations.

We performed *ab initio* molecular dynamics (AIMD) simulations to study diffusion mechanism in the model SIC materials, LGPS, cubic-phase Li_7_La_3_Zr_2_O_12_ (LLZO) and Li_1.3_Al_0.3_Ti_1.7_(PO_4_)_3_ (LATP) (Fig. 2 and Supplementary Fig. 1), which have different anion packing (that is, bcc in LGPS versus non-bcc in LLZO and LATP). The high Li ionic conductivities and low activation energies calculated from AIMD simulations are in good agreement with experimental values^7^,^9^,^11^ (Supplementary Table 1 and Supplementary Fig. 1). By analysing Li^+^ dynamics from AIMD simulations, we found that most Li ions migrate in a highly concerted fashion, that is, multiple ions hop simultaneously into their nearest sites within a few picoseconds (Supplementary Note 1 and Supplementary Fig. 2). The strong time correlation in Li^+^ hopping during the concerted migration is confirmed by the van Hove correlation function (Fig. 2g–i) of Li^+^ dynamics. In addition, to characterize the extent of concerted migrations, we calculated the correlation factor related to the Haven ratio. Whereas a correlation factor of 1.0 corresponds to isolated single-ion diffusion, the correlation factor is calculated as 3.0, 3.0 and 2.1 for LGPS, LLZO and LATP, respectively, in the AIMD simulations at 900 K, corresponding to correlated hopping of approximately two to three ions on average in these SICs. Therefore, the concerted migration is the dominant mechanism for fast diffusion in SICs, as it is in liquids^19^,^20^.

**Figure 3:**
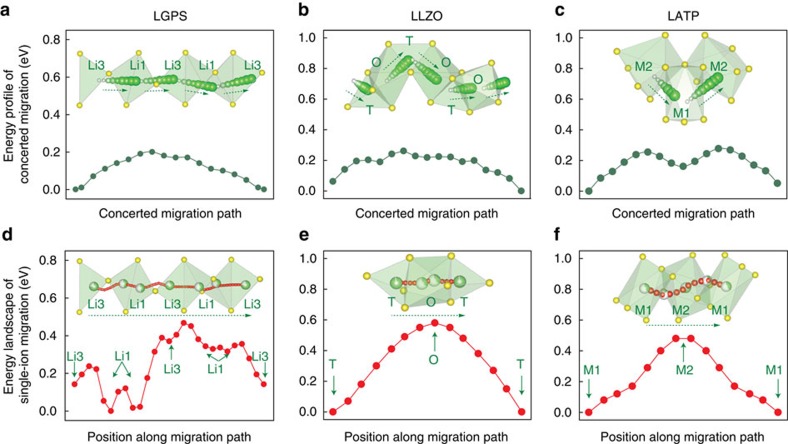
Concerted migration and energy landscape in super-ionic conductors. (**a**–**c**) Migration energy barrier in (**a**) LGPS, (**b**) LLZO, (**c**) LATP for concerted migration of multiple Li ions hopping into the next sites along the diffusion channel. Insets show the Li^+^ path (green spheres) and O/S ions (yellow spheres). (**d**–**f**) The energy landscape of single Li^+^ along the migration channel (shown in insets) across multiple Li sites (partially filled green sphere) and Li^+^ pathway (red spheres).

The concerted migration extracted from AIMD simulations (illustrated as insets Fig. 3a–c) is simultaneous hopping of multiple adjacent ions into their nearest sites. In LGPS, a typical concerted migration involves four Li ions occupying Li1 and Li3 sites hopping simultaneously along the *c* channel into their nearest-neighbour Li3 and Li1 sites, respectively (Fig. 3a), as observed in a previous study^21^. In LLZO, Li ions partially co-occupy tetrahedral (T) sites and octahedral (O) sites. During concerted migration in LLZO, T-site Li ions hop to the nearest-neighbour O sites and the Li ions occupying these O sites hop into their nearest neighbour T sites, resulting in concerted hopping of multiple Li ions along the garnet diffusion channel (Fig. 3b) similar to previous modelling studies^22^,^23^. In LATP, the typical concerted migration mode is that two Li ions at adjacent M1 and M2 sites migrate in pair. The Li^+^ on the M1 site hops into the unoccupied M2 site, and at the same time the Li^+^ on the M2 site hops into the next M1 site (Fig. 3c). The migration barriers of these concerted migrations were calculated using nudged-elastic-band (NEB) methods based on *ab initio* computation (Fig. 3a–c), and were found to be 0.20, 0.26 and 0.27 eV in LGPS, LLZO and LATP, respectively. Given the highly disordered nature of the Li sublattice, various modes of concerted migration mechanisms involving different number of Li and different Li configurations were observed during AIMD simulations and are illustrated in Supplementary Note 2 and Supplementary Fig. 3. The other modes of concerted migration also show migration barriers similar to the typical modes in Fig. 3. The calculated energy barriers of concerted migration are in good agreement with the activation energies obtained from the AIMD simulations and from experiments^7^,^9^,^11^ (Supplementary Table 1). Therefore, these typical concerted migrations observed in AIMD simulations represent the key diffusion mechanisms in SICs.

### Origin of concerted migration with low barriers

Given such low energy barriers for multi-ion concerted migration, a relatively flat energy landscape along Li^+^ diffusion channels is expected. Surprisingly, the energy landscapes have barriers of 0.47, 0.58 and 0.49 eV for LGPS, LLZO and LATP, respectively (Fig. 3d–f), which are significantly higher than the energy barrier of concerted migration. On the basis of the classical diffusion model, these high barriers of the energy landscape would lead to even higher activation energy *E*_a_, as each migrating ion feels the high barriers of the energy landscape along the diffusion channel. Thus, the low-barrier concerted migration of multiple ions cannot be explained by the classical diffusion model. As super-ionic conduction is only activated at specific composition with high Li concentration, the mobile-ion configuration and the interactions among these ions, which are neglected in the classical diffusion model, must be considered in order to properly describe the concerted migration in SICs.

**Figure 4:**
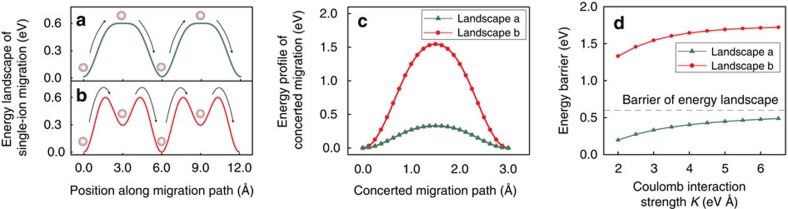
Diffusion model for concerted migration. (**a**,**b**) The potential energy of the structural framework with low (**a**) or high (**b**) barriers at the high-energy sites. The mobile ion (grey sphere) configurations and the migration paths (arrows) are illustrated. (**c**) The energy profile for the concerted migration in the energy landscape (**a**) and (**b**) at *K*=3 eV Å. (**d**) The energy barrier of concerted migration at different Coulomb interaction strength *K*.

To reveal the mechanism of multi-ion concerted migration, here we performed a simple diffusion model on the basis of the classical diffusion model by taking into account the configuration of mobile ions and Coulomb interactions among them. In this model, we chose an energy landscape (Fig. 4a) with a 0.6 eV barrier, similar to that in LLZO (Fig. 3e), and included Coulomb interaction among mobile ions with a strength *K* of ∼2–4 eV Å fitted to *ab initio* calculations (Supplementary Note 3). In addition, the unique Li^+^ configuration in SIC materials (Fig. 3d–f) was also considered in this model. In SICs, the mobile ions occupy the high-energy sites (Fig. 4a,b), such as the octahedral O sites in LLZO (Fig. 3e) and the M2 sites in LATP (Fig. 3f), which are near the highest energy point along the diffusion path. At high Li concentration of these SIC materials, the high-energy sites in SICs are occupied because the low-energy sites (for example, tetrahedral T sites in LLZO and M1 sites in LATP) are preferably occupied and cannot accommodate all Li ions inserted. The extra Li ions occupying high-energy sites are stabilized by Coulomb interactions from nearby mobile ions (within ∼2–3 Å) during the minimization of the overall lattice energy.

Our model shows that such a unique mobile-ion configuration under strong mobile ion-ion interactions is the key for achieving low-barrier concerted migration in these SICs. At typical *K* values of 2–4 eV Å in these SICs, the concerted migration of multiple ions shows a significantly lower migration barrier of ∼0.2–0.4 eV (Fig. 4d and Supplementary Fig. 4), which is in good agreement with those from NEB calculations (Fig. 3a–c) and AIMD simulations (Supplementary Table 1). Therefore, this simple diffusion model captures the key physics of concerted migration in the SICs. This model demonstrates that low energy barrier of multi-ion concerted migration is a result of the unique mobile-ion configuration with high-energy site occupancy. During the concerted migration of multiple ions, the ions located at the high-energy sites migrate downhill, which cancels out a part of the energy barrier felt by other uphill-climbing ions. As a result, concerted migration of multiple ions has a significantly lower energy barrier than the energy landscape of the crystal structural framework.

In addition, high-energy sites should have locally low barriers and flat energy landscapes, in order to activate low-barrier concerted migration. As observed in the AIMD simulations (Fig. 2d,e), the high-energy sites are associated with elongated spatial occupancy density of mobile Li ions. For example, the Li probability density is elongated at the octahedral (O) sites in LLZO (Fig. 2e). That elongated density indicates a locally flat energy landscape for Li^+^ to hop out. The easy migration of ions occupying high-energy sites may facilitate the onset of multi-ion concerted migration^24^. Otherwise, for the energy landscape in Fig. 4b as in non-SIC materials (Supplementary Note 4 and Supplementary Fig. 5), multiple ions would simultaneously climb uphill, leading to higher energy barrier for concerted migration (Fig. 4c).

## Discussion

In summary, our theory demonstrates a simple conceptual framework for understanding fast ion diffusion in SICs. Specifically, mobile ions occupying high-energy sites can activate concerted migration with a reduced migration energy barrier. In addition to lithium garnet and NASICON SICs, this mechanism is observed in other SIC materials, such as Li_7_P_3_S_11_, β-Li_3_PS_4_, LISICON, Li_*x*_La_2/3−*x*/3_TiO_3_ (LLTO) perovskite^25^, and Na^+^-conducting NASICON, where high-energy sites are occupied along the diffusion path and the concerted migration with low energy barrier is confirmed in *ab initio* modelling (Supplementary Note 5 and Supplementary Figs 6–11). The concerted migration of multiple ions is also reported for low-barrier diffusion in other Li ionic conductors, for example, Li_3_OX (X=Cl, Br)^26^ and doped Li_3_PO_4_ (refs 27, 28). In addition to Li^+^ and Na^+^ conductors, our proposed model is generally applicable to conductors of other ions. For example, Ag super-ionic conductor AgI^29^ is known for highly concerted migration, and oxygen ionic conductors of fluorite structure (for example, Bi_2_O_3_) (Supplementary Fig. 12) and La_1−*x*_Ba_1+*x*_GaO_4−*x*/2_ (ref. 30) also show concerted migration behaviour. Therefore, our proposed theory and identified mechanism are universally applicable to fast diffusion in a broad range of ion-conducting materials.

Moreover, our theory provides a simple strategy for designing super-ionic conductor materials, that is, inserting mobile ions into high-energy sites to activate concerted ion migration with lower barriers. This explains how super-ionic conduction in lithium garnet and NASICON SICs is activated at certain compositions with increased Li concentration. Here, we demonstrate this strategy by designing a number of novel fast ion conducting materials to activate concerted migration with reduced diffusion barrier. We select LiTaSiO_5_ and LiAlSiO_4_ (details of structures in Supplementary Note 6 and Supplementary Figs 13 and 14), which have structures with a decent bottleneck size of diffusion channels and well-connected Li^+^ percolation network, but have not been studied for Li^+^ transport. The original structures show low Li^+^ conductivities and high activation energies similar to their high-barrier energy landscapes (Supplementary Figs 13 and 14). Extra Li ions are inserted into the high-energy sites of LiTaSiO_5_ and LiAlSiO_4_ by aliovalent substitution of non-Li cations with lower valences. For the doped materials, AIMD simulations show Li^+^ concerted migrations with significantly reduced migration barriers of 0.23–0.28 eV and Li^+^ conductivities of 1–4 mS cm^−1^ at RT (Supplementary Figs 13 and 14), which are comparable to many known Li SICs. These results demonstrate that the design strategy based on our simple conceptual framework can be successfully utilized to design novel fast ion conducting materials. In addition, this strategy for facilitating diffusion is generally applicable to any ion-conducting materials.

## Methods

### Density functional theory computation

All density functional theory (DFT) calculations in this study were performed using Vienna *Ab initio* Simulation package (VASP)^31^ within the projector augmented-wave approach. Perdew–Burke–Ernzerhof (PBE)^32^ generalized-gradient approximation (GGA) functionals were adopted in all calculations. The parameters in static DFT calculations were consistent with the Materials Project^33^,^34^,^35^. The nudged elastic band (NEB) calculations were performed in supercell models using a *Γ*-centred 2 × 2 × 2 *k*-point grid.

### *Ab initio* molecular dynamics simulation

*Ab initio* molecular dynamics (AIMD) simulations were performed in supercell models using non-spin-polarized DFT calculations with a Γ-centred *k*-point. The time step was set to 2 fs. The initial structures were statically relaxed and were set to an initial temperature of 100 K. The structures were then heated to targeted temperatures (300–1500 K) at a constant rate by velocity scaling over a time period of 2 ps. The NVT ensemble using a Nosé–Hoover thermostat^36^ was adopted. The total time of AIMD simulations were in the range of 100 to 600 ps until the total mean square displacement of Li ions was >250 Å^2^ in each AIMD simulation and until the diffusivity was converged.

As in previous studies^37^,^38^,^39^, the diffusivity *D* was calculated as the mean square displacement over time interval Δ*t*:


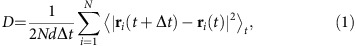


where *d*=3 is the dimension of the diffusion system, *N* is the total number of diffusion ions, **r**_*i*_(*t*) is the displacement of the *i*-th ion at time *t*, and the bracket represents averaging over *t*. The ionic conductivity was calculated based on the Nernst-Einstein relationship using


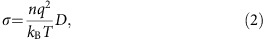


where *n* is the number of mobile ions per unit volume and *q* is the ionic charge. The probability density of mobile ions was calculated as the fraction of time that each spatial location was occupied.

### Time correlation of Li^+^ dynamics

The van Hove correlation function^40^ was calculated from the AIMD simulations. The distinctive part *G*_d_ describes the radial distribution of different ions after time interval Δ*t* with respect to the initial ion,


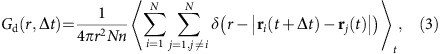


where *δ* is the Dirac delta function. The correlation function is averaged over the time *t*.

The Haven ratio is often used to measure the correlation effect in ionic diffusion^41^. In this study, we defined a similar correlation factor *f* to quantify the correlation of ion migration:


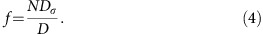


While *D* is the self-diffusion diffusivity of individual ions, *D*_*σ*_ is the diffusivity of the centre of all diffusion ions and is calculated as:


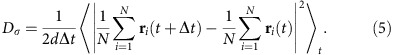


### Energy landscape of single-ion migration

The energy landscape of a single Li^+^ along the migration channel (Fig. 3d–f) was calculated using the NEB methods. In LGPS, three Li^+^ ions in the *c* channel were removed and the energy landscape was obtained by migrating a Li^+^ across the *c* channel. The energy landscape of cubic-phase LLZO corresponds to single Li^+^ migration between two neighbouring tetrahedral sites after removing a Li ion. The energy landscape of LATP corresponds to single Li^+^ migration between two neighbouring M1 sites after removing a Li ion. In the NEB calculations, the charge states of all ions were maintained by inserting extra electrons into the system as in the previous study^13^. To avoid excessive relaxation of the Li^+^ sublattice, only non-Li cations and anions were relaxed during the NEB calculations.

### Diffusion model for concerted migration

In the diffusion model illustrated in Fig. 4, four mobile ions were arranged in a 1D lattice consisting of two unit cells. The 1D unit cell has a period of *L*=6 Å, which is similar between two nearest-neighbour M1 sites in LATP (Figs 2f and 3f). The total energy *E* of the entire mobile lattice is given by the sum of the potential energy *ϕ* from the crystal framework (that is, the energy landscape) and the Coulomb interaction among mobile ions:


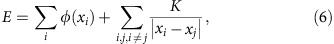


where *x*_*i*_ is the position of the ion *i* and *K* is the Coulomb interaction strength between two mobile ions. The lattice energy landscape *ϕ* considered in the main text (Fig. 4a,b) is defined as follows. The energy landscape in Fig. 4a is given by


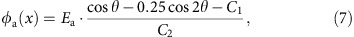


where *θ*=2π*x*/*L*−π and the normalization factors *C*_1_ and *C*_2_ are −1.25 and 2.00, respectively. The highest point of the energy landscape is *E*_a_=0.6 eV, which is set similar to the single Li-ion energy landscape of LLZO (Fig. 3e). The energy landscape in Fig. 4b is given by


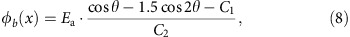


where *C*_1_=−2.50 and *C*_2_=4.08. This energy landscape has the same highest energy point of 0.6 eV, but has a higher local barrier of 0.3 eV at the high-energy sites (Fig. 4b).

### Materials

The crystal structures investigated were obtained from the Inorganic Crystal Structure Database^42^ and Materials Project^35^. The structures with disordered site occupancies were ordered using the same method used in previous studies^38^,^39^. The structure of LATP was derived from the LiTi_2_(PO_4_)_3_ structure by partially substituting Ti with Al and by inserting extra Li atoms into M2 sites (Fig. 2f). The occupancy of Al/Ti and Li were ordered to obtain the structure.

### Data availability

The computation data to support the findings of this study is available from the corresponding author on reasonable request.

## Additional information

**How to cite this article:** He, X. *et al*. Origin of fast ion diffusion in super-ionic conductors. *Nat. Commun.* **8**, 15893 doi: 10.1038/ncomms15893 (2017).

**Publisher’s note:** Springer Nature remains neutral with regard to jurisdictional claims in published maps and institutional affiliations.

## Supplementary Material

Supplementary Information

## Figures and Tables

**Figure 1:**
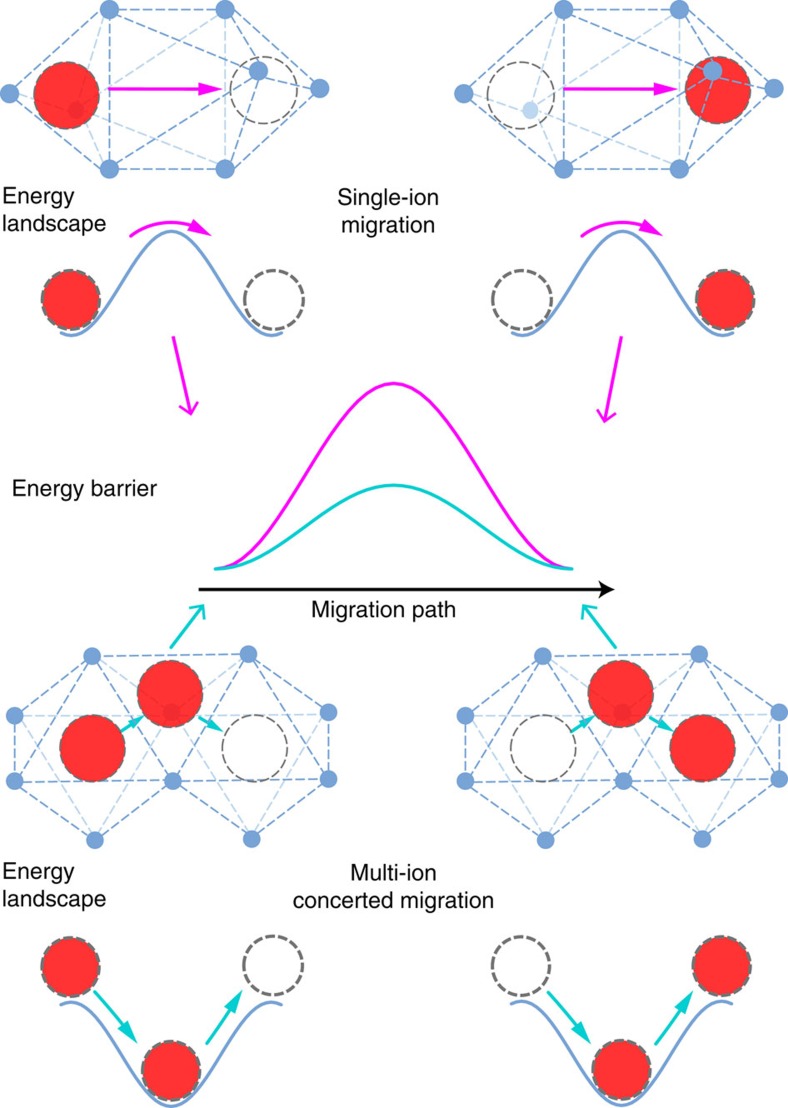
Schematic illustration of single-ion migration versus multi-ion concerted migration. For single-ion migration (upper insets), the migration energy barrier is the same as the barrier of the energy landscape. In contrast, the concerted migration of multiple ions (lower insets) has a lower energy barrier as a result of strong ion-ion interactions and unique mobile ion configuration in super-ionic conductors.
