# Supplementary material for: Origin of fast ion diffusion in super-ionic conductors
Source: Nat Commun. 2017 Jun 21;8:15893. doi: 10.1038/ncomms15893 (PMC5482052; doi:10.1038/ncomms15893)
Supplement: Supplementary Information [file ncomms15893-s1.pdf]

File name: Supplementary Information
Description: Supplementary Figures, Supplementary Tables, Supplementary Notes and Supplementary References

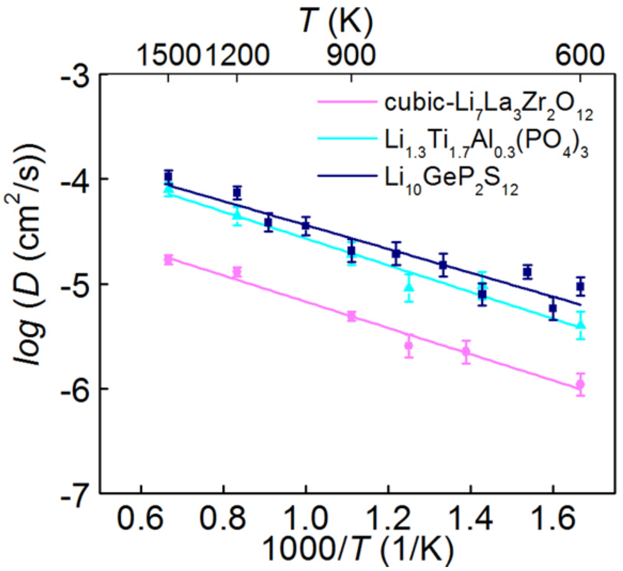

Supplementary Figure 1. Diffusion properties of super-ionic conductors. Arrhenius plot of Li^+ diffusivity *D* in $Li_{10}GeP_2S_{12}$, cubic-$Li_7La_3Zr_2O_{12}$, $Li_{1.3}Ti_{1.7}Al_{0.3}(PO_4)_3$ from AIMD simulations. The error bar of *D* is the statistical uncertainty from the linear fitting of mean square displacement (MSD) over time[8].

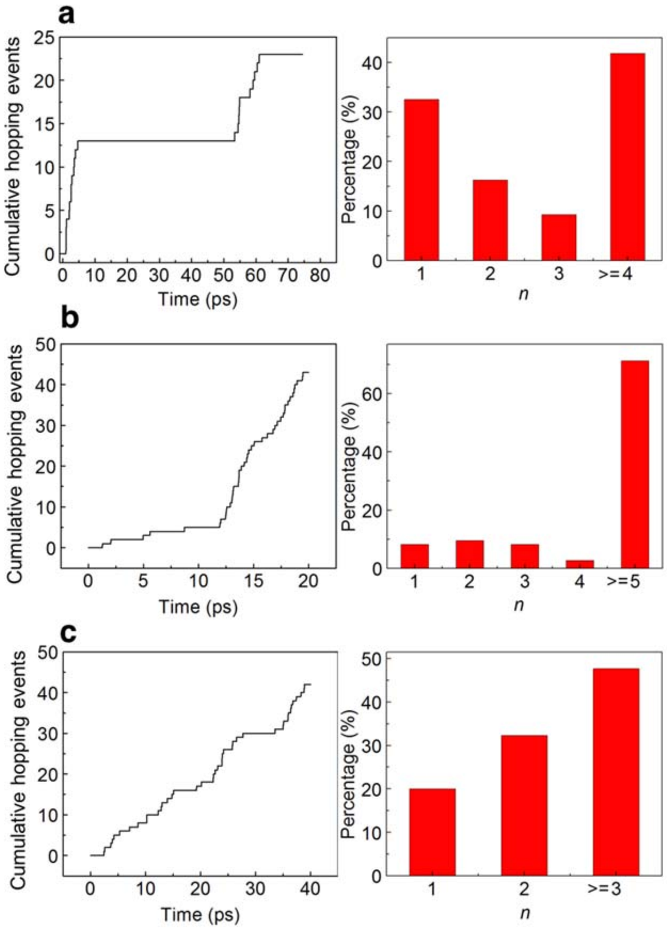

Supplementary Figure 2. Statistical analysis of hopping events. Cumulative hopping events over time (left panels) and the histograms of the number *n* of Li^+ hopping (right panels) from AIMD simulations of (**a**) LGPS at 300 K, (**b**) cubic-phase LLZO at 600 K, (**c**) LATP at 600 K.

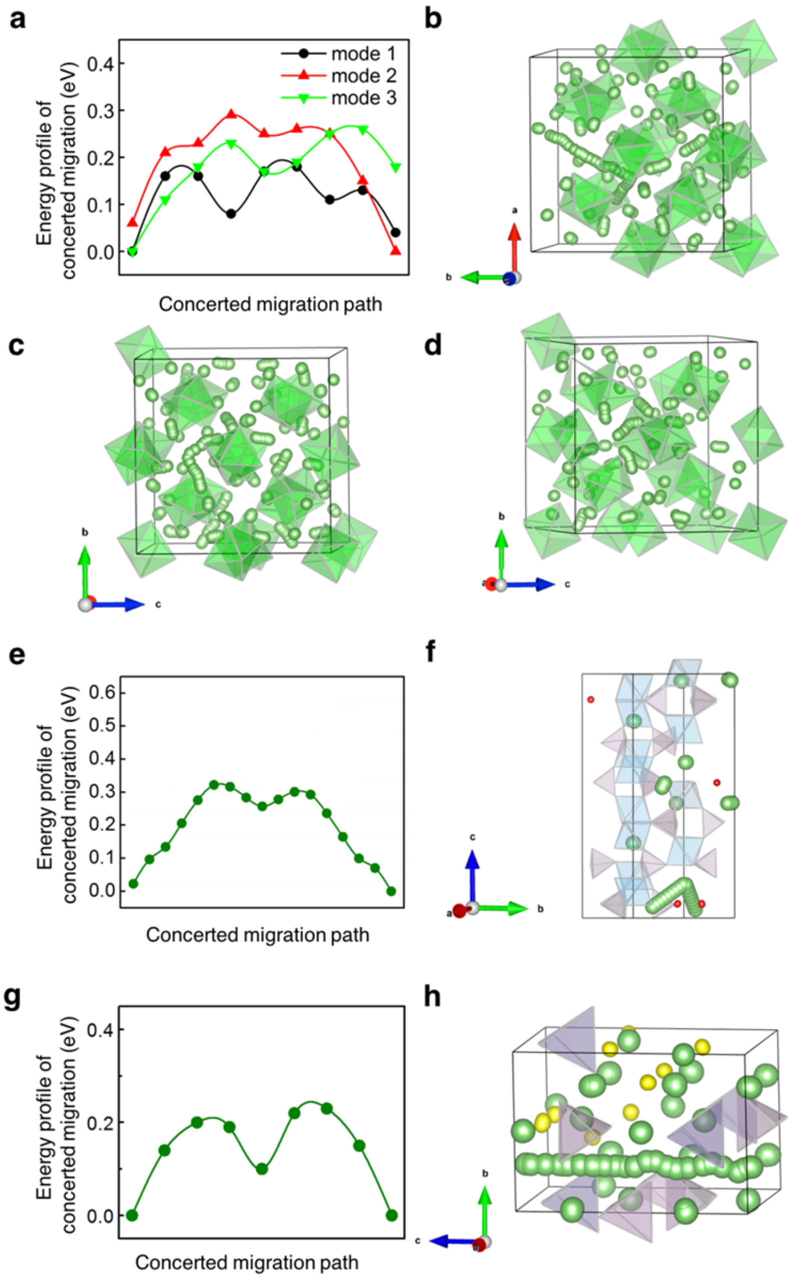

Supplementary Figure 3. Other concerted migration mechanism in SICs. Energy profile and migration paths of Li^+ concerted migration with different migration modes in (**a**–**d**) cubic-phase LLZO at 600 K, (**e**–**f**) LATP at 600 K, (**g**–**h**) LGPS at 400 K. The migration paths (green spheres) are merged from NEB images.

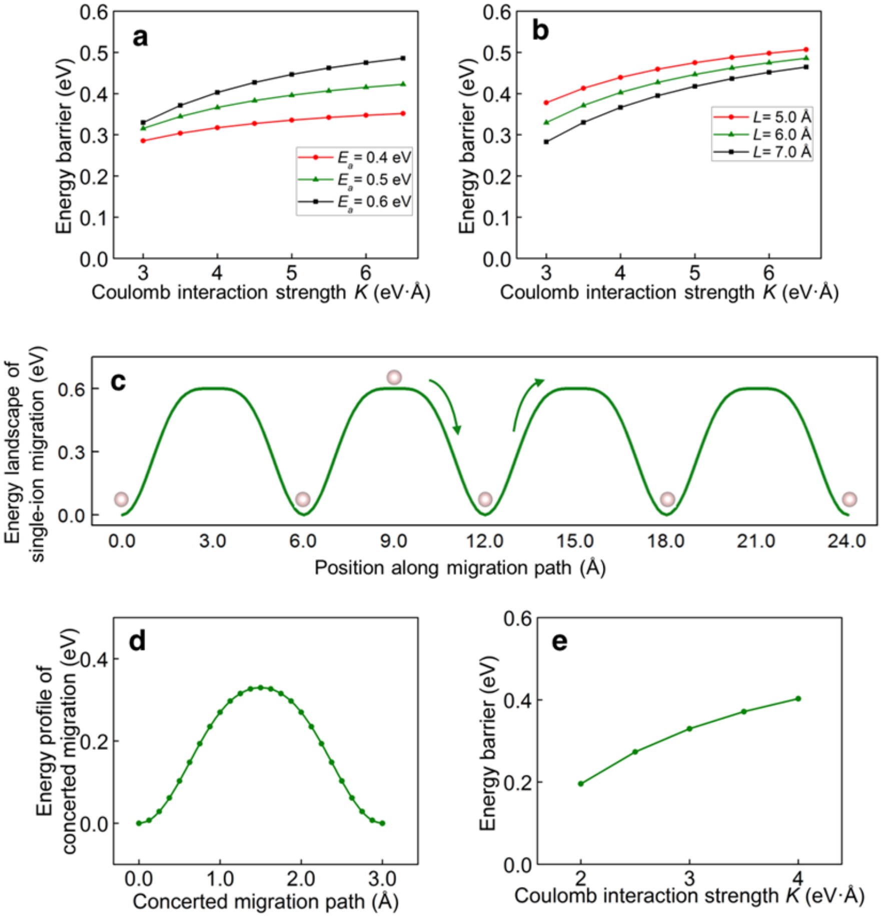

Supplementary Figure 4. Diffusion model for concerted migration. (a, b) The energy barrier of concerted migration as a function of the Coulomb interaction strength K in the energy landscape (Fig. 4a) with (**a**) different E_a at L = 6 Å or (**b**) with different L at E_a = 0.6 eV. **(c)** Diffusion model with a different ion configuration in the energy landscape of Fig. 4a. The mobile ion (grey sphere) configuration and the concerted migration path (arrows) are illustrated. **(d)** The energy profile for the concerted migration at K = 3 eV·Å. **(e)** The energy barrier for concerted migration as a function of the Coulomb interaction strength K.

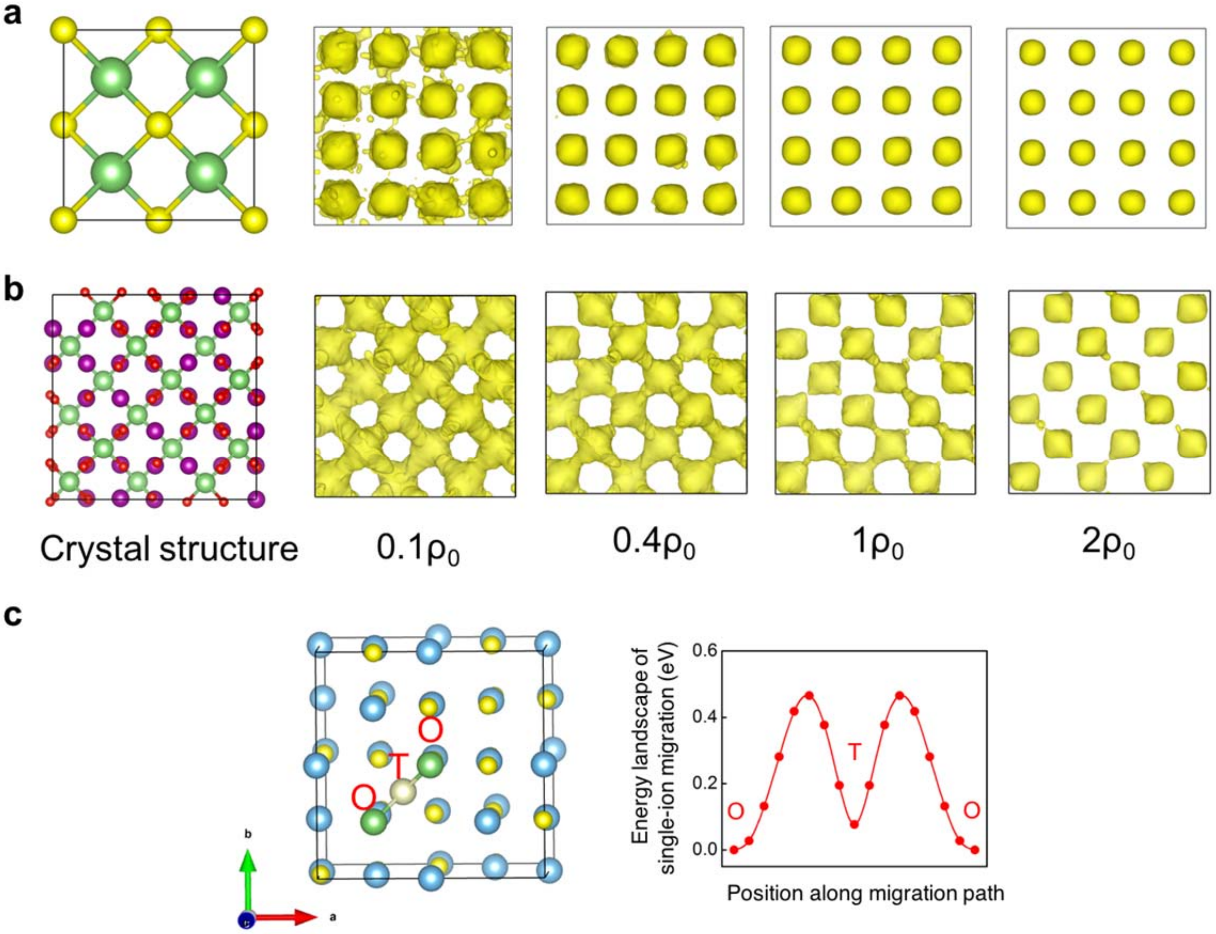

Supplementary Figure 5. Li ion diffusion in non-SICs. (a, b) Crystal structure (left) for (**a**) Li_2S and (**b**) $LiMn_2O_4$, and Li^+ probability densities at different isosurfaces (right), where ρ_0 is the average probability density. **(c)** Crystal structure (left) and the energy landscape (right) of single Li^+ migration in $LiTiS_2$. Li, Mn, Ti, S and O are represented by green, purple, blue, yellow and red spheres, respectively. Octahedral (O) and tetrahedral (T) sites in $LiTiS_2$ are marked as dark and light green spheres, respectively.

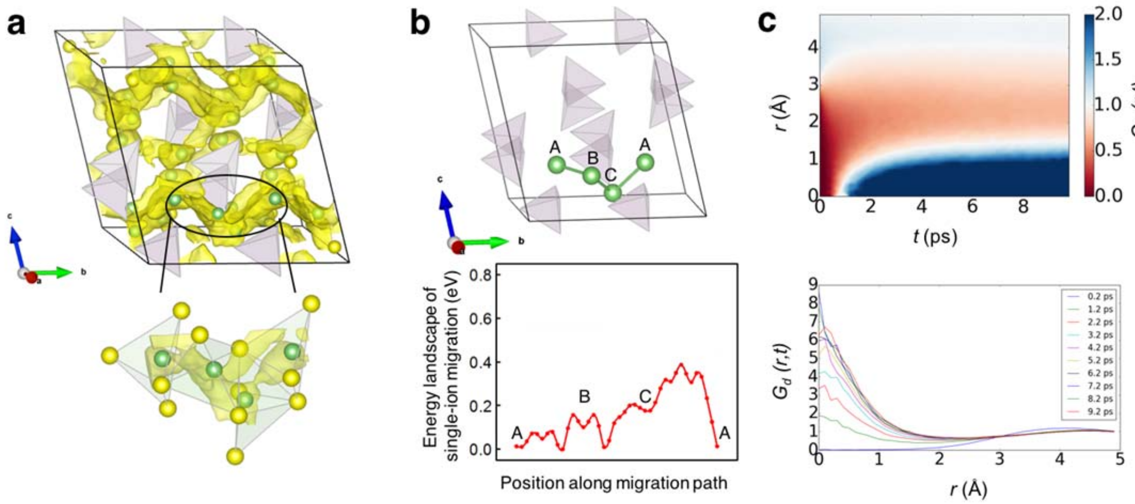

Supplementary Figure 6. Li ion diffusion in $Li_7P_3S_{11}$. **(a)** Li^+ probability density from AIMD at 900 K. The isosurfaces are plotted at $4\rho_0$, whereas ρ_0 is the average density. Li^+, S^{2-}, and PS_4 polyhedra are colored as green, yellow, and purple, respectively. **(b)** Energy landscape of single Li^+ migration along typical diffusion paths. The high-energy sites are partially occupied in these materials. **c**, Distinctive part of van Hove correlation function for Li ions at 900 K.

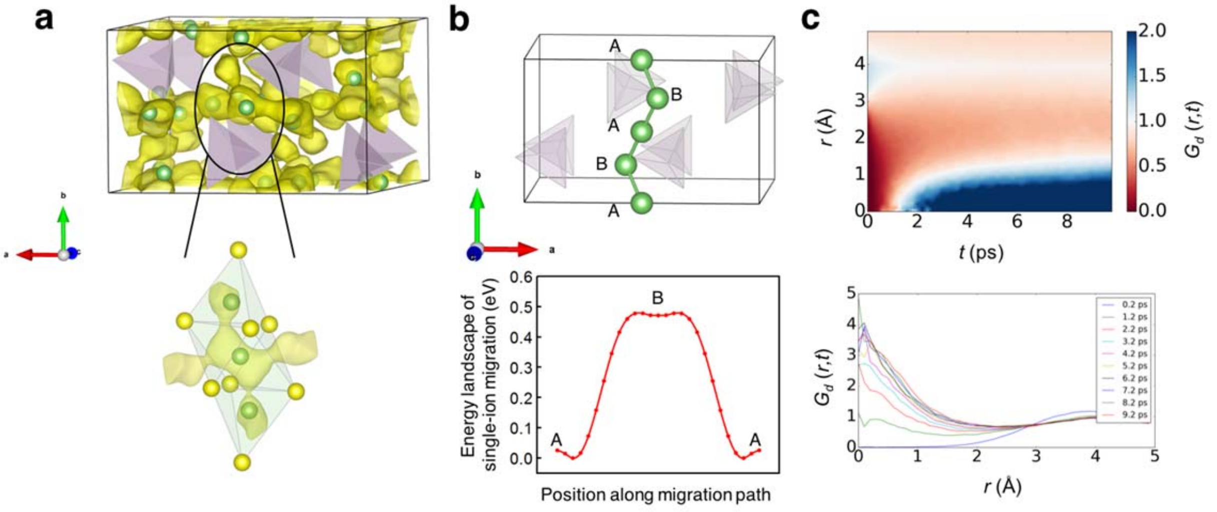

Supplementary Figure 7. Li ion diffusion in β-Li_3PS_4. **(a)** Li^+ probability density from AIMD at 900 K. The isosurfaces are plotted at $4\rho_0$, whereas ρ_0 is the average density. Li^+, S^{2-}, and PS_4 polyhedra are colored as green, yellow, and purple, respectively. **(b)** Energy landscape of single Li^+ migration along typical diffusion path. The high-energy sites are partially occupied in these materials. **(c)** Distinctive part of van Hove correlation function for Li ions at 900 K.

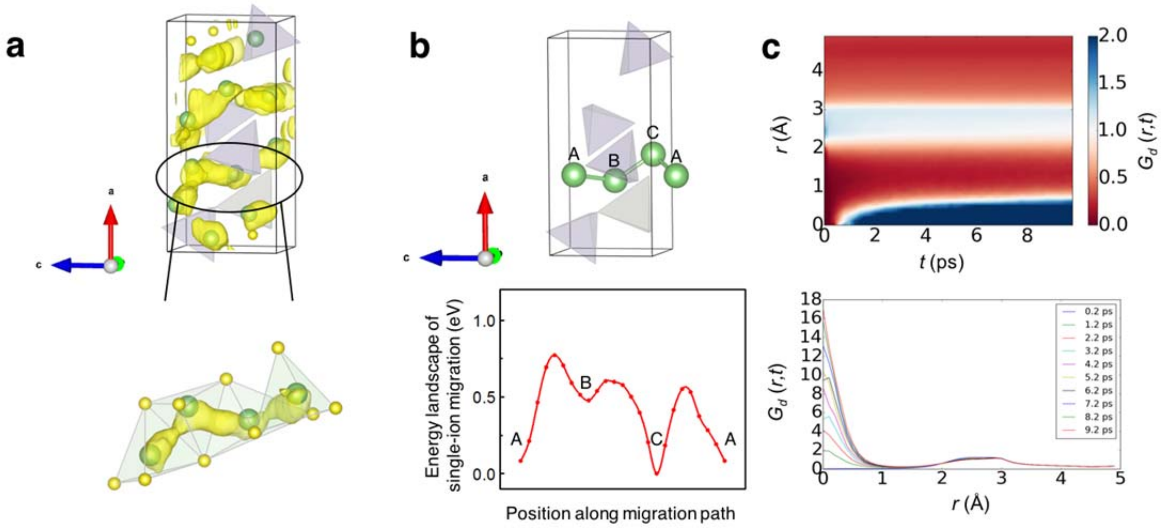

Supplementary Figure 8. Li ion diffusion in $Li_{14}ZnGe_4O_{16}$. (a) Li^+ probability density from AIMD at 900 K. The isosurfaces are plotted at $4\rho_0$, whereas ρ_0 is the average density. Li^+, O^{2-}, and GeO_4 polyhedra are colored as green, yellow, and purple, respectively. **(b)** Energy landscape of single Li^+ migration along typical diffusion paths. Since Zn shares sites with Li, we removed Zn and kept GeO_4 at original position in our calculation of energy landscape. The high-energy sites are partially occupied in these materials. **(c)** Distinctive part of van Hove correlation function for Li ions at 900 K.

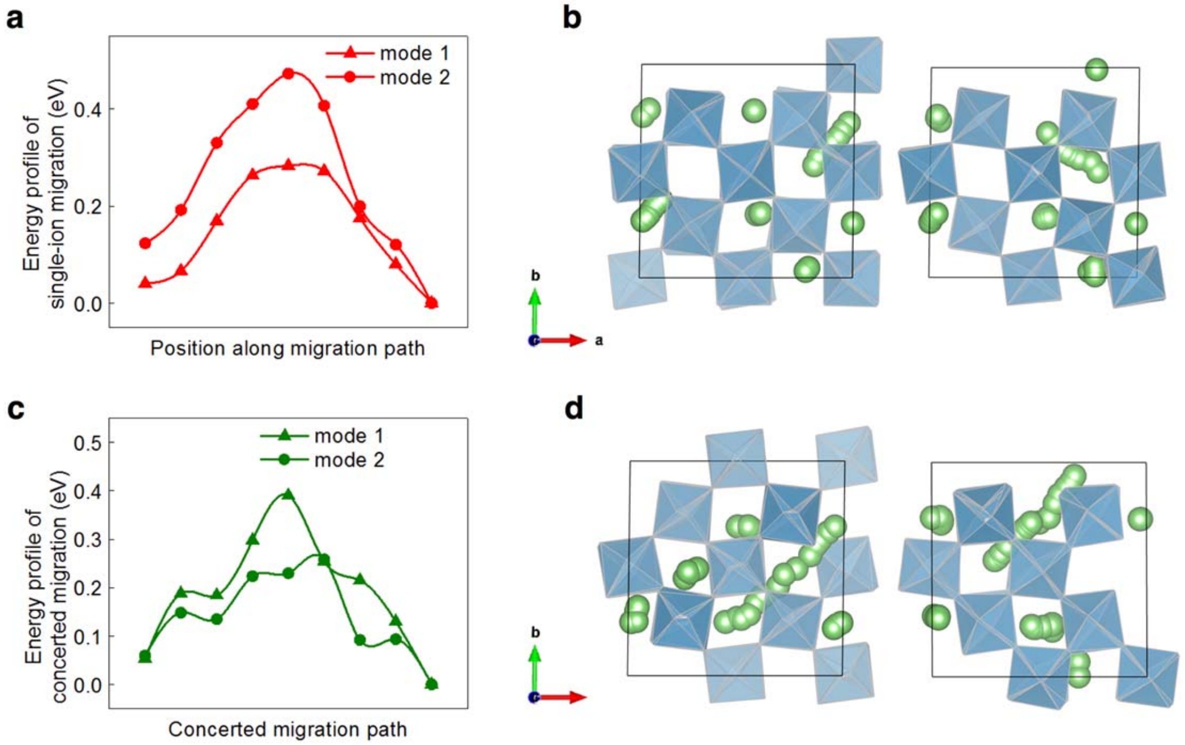

Supplementary Figure 9. Li ion diffusion in $Li_{0.31}La_{0.56}TiO_3$ (LLTO). (a, b) Energy profile and migration path of single Li^+ migration along typical diffusion paths. **(c, d)** Energy profile and migration path of concerted migration with two neighboring Li^+ in LLTO. The migration paths (green spheres) are merged from NEB images. TiO_6 polyhedra are colored as blue and La ions are not shown for clarity.

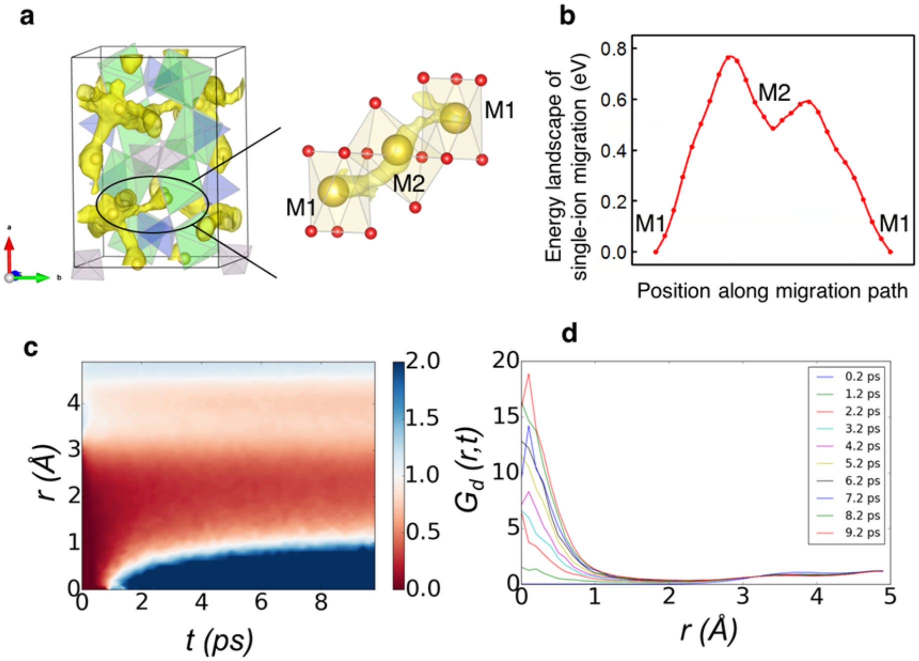

Supplementary Figure 10. Na ion diffusion in NASICON. (a) Na^+ probability density in $Na_3Zr_2Si_2PO_{12}$ from AIMD simulation at 900 K. The isosurface is plotted at $4\rho_0$, where ρ_0 is the average density. **(b)** Energy landscape of single Na^+ migration along typical diffusion paths. **(c, d)** Distinctive part of van Hove correlation function for Na^+ in $Na_3Zr_2Si_2PO_{12}$ at 900 K.

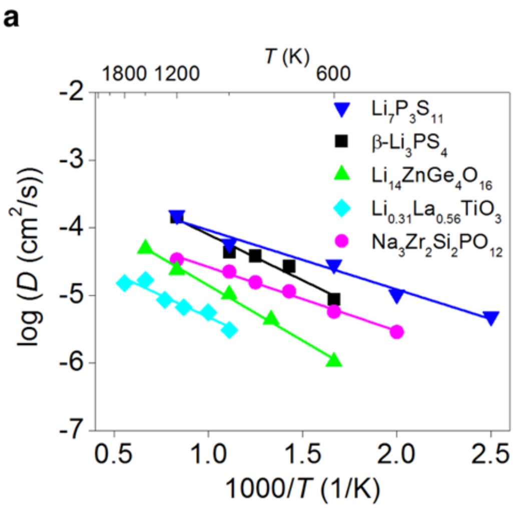

b

Composition	E_a (eV)
$Li_7P_3S_{11}$ (AIMD)	0.17 ± 0.01
$Li_7P_3S_{11}$ (Expt.)[9]	0.18 (~300 K)
β-Li_3PS_4 (AIMD)	0.27 ± 0.03
β-Li_3PS_4 (Expt.)[10]	0.16 (~500 K)
$Li_{14}ZnGe_4O_{16}$ (AIMD)	0.32 ± 0.01
$Li_{14}ZnGe_4O_{16}$ (Expt.)[11,12]	0.4-0.6 (~500 K)
$Li_{0.31}La_{0.56}TiO_3$ (AIMD)	0.26 ± 0.04
$Li_{0.33}La_{0.56}TiO_3$ (Expt.)[11,13]	0.26-0.40
$Na_3Zr_2Si_2PO_{12}$ (AIMD)	0.19 ± 0.01
$Na_3Zr_2Si_2PO_{12}$ (Expt.)[15]	0.20-0.24 (>450 K)

Supplementary Figure 11. Summary of diffusion properties in other super-ionic conductors. **(a)** Arrhenius plot of Li^+/Na^+ diffusivity *D* from AIMD simulations. **(b)** Calculated (AIMD) and experimental (Expt.) activation energy E_a.

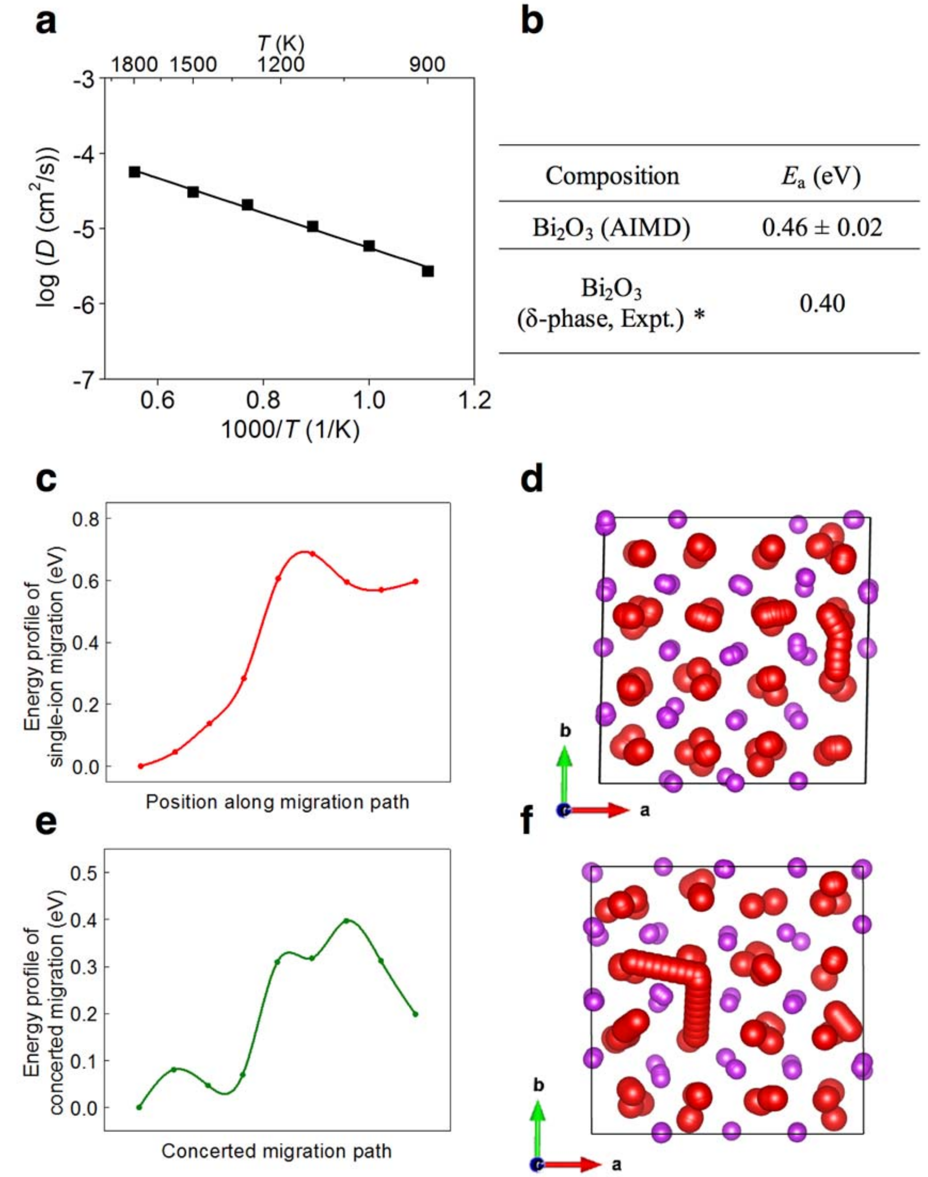

Supplementary Figure 12. Oxygen ion diffusion in Bi_2O_3. (a) Arrhenius plot of O^{2-} diffusivity D in Bi_2O_3 from AIMD simulations. **(b)** Calculated (AIMD) and experimental (Expt.) O^{2-} activation energy E_a. **(c, d)** Energy profile and migration path of single O^{2-} migration along typical diffusion paths. **(e, f)** Energy profile and migration path of concerted migration with two O^{2-} in Bi_2O_3 at 750 K. The migration paths (red spheres) are merged from NEB images. Bi and O ions are colored as purple and red, respectively.

* Ref. 16

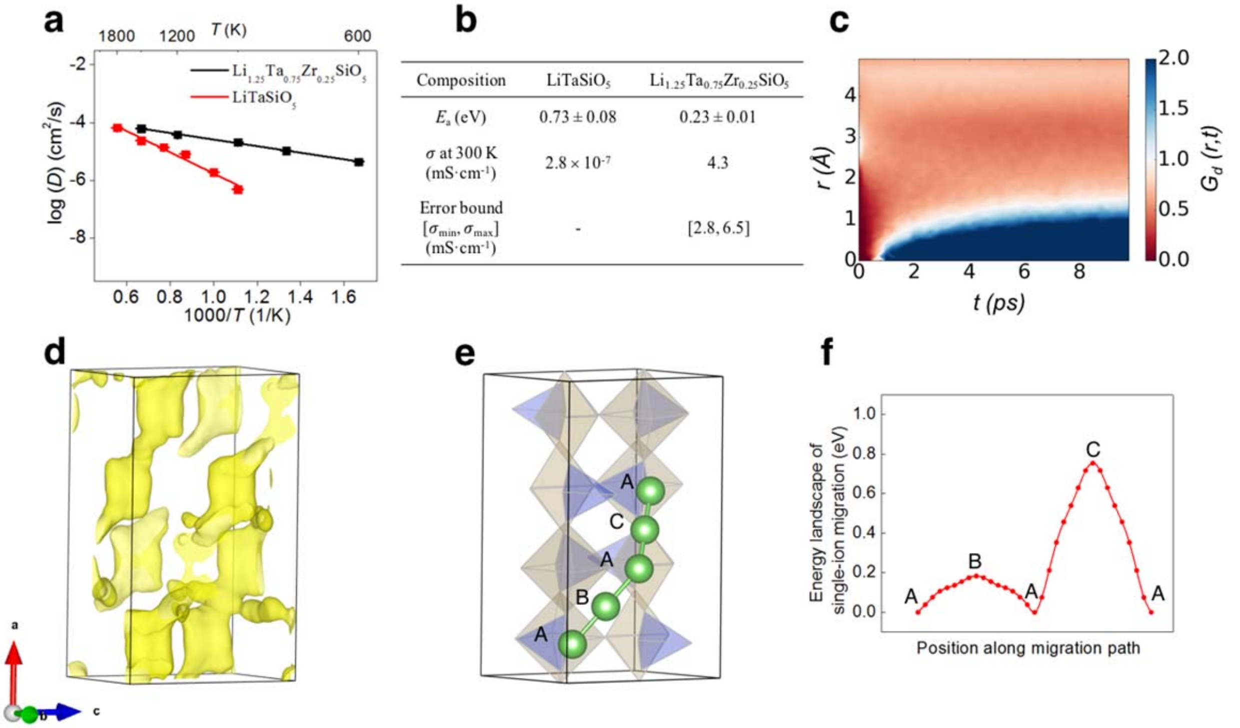

Supplementary Figure 13. Li ion diffusion in $Li_{1+x}Ta_{1-x}Zr_xSiO_5$ (x = 0, 0.25). (a) Arrhenius plot of Li^+ diffusivity D in $Li_{1+x}Ta_{1-x}Zr_xSiO_5$ (x = 0 and 0.25) from AIMD simulations. **(b)** Calculated Li^+ conductivity σ at 300 K and activation energy E_a. **(c)** Distinctive part of van Hove correlation function for Li^+ in $Li_{1.25}Ta_{0.75}Zr_{0.25}SiO_5$ at 900 K. **(d)** Li^+ probability density in $Li_{1.25}Ta_{0.75}Zr_{0.25}SiO_5$ from AIMD simulation at 900 K. The isosurface is plotted at $4\rho_0$, whereas ρ_0 is the average density. **(e)** Crystal structure of $LiTaSiO_5$. Li sites and MO_x (M = Ta, Si) polyhedra are colored as green, yellow, and purple, respectively. High-energy B and C sites are partially occupied in doped $Li_{1.25}Ta_{0.75}Zr_{0.25}SiO_5$. **(f)** Energy landscape of single Li^+ migration along typical diffusion paths.

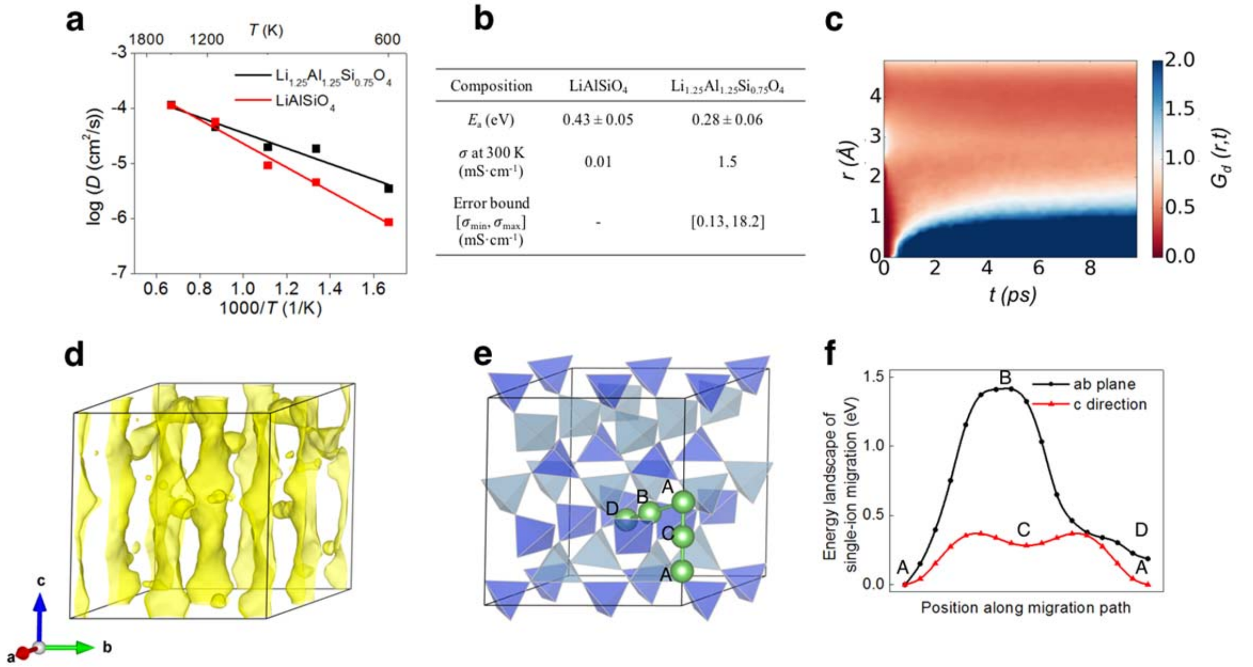

Supplementary Figure 14. Li ion diffusion in $Li_{1+x}Al_{1+x}Si_{1-x}O_4$ (x = 0, 0.25). (a) Arrhenius plot of Li^+ diffusivity D in $Li_{1+x}Al_{1+x}Si_{1-x}O_4$ (x = 0, 0.25) from AIMD simulations. (b) Calculated Li^+ conductivity σ at 300 K and activation energy E_a. (c) Distinctive part of van Hove correlation function for Li^+ in $Li_{1.25}Al_{1.25}Si_{0.75}O_4$ at 900 K. (d) Li^+ probability density in $Li_{1.25}Al_{1.25}Si_{0.75}O_4$ from AIMD simulation at 900 K. The isosurface is plotted at ρ_0, whereas ρ_0 is the average density. (e) $LiAlSiO_4$ crystal structure. Li sites and MO_x (M = Al, Si) polyhedra are colored as green, grey, and purple, respectively. High-energy B and C sites are partially occupied in doped $Li_{1.25}Al_{1.25}Si_{0.75}O_4$. (f) Energy landscape of single Li^+ migration along typical diffusion paths.

Supplementary Table 1. Calculated (AIMD) and experimental (Expt.) Li^+ conductivity σ and activation energy E_a of $Li_{10}GeP_2S_{12}$ (LGPS), $Li_7La_3Zr_2O_{12}$ (LLZO) and $Li_{1.3}Ti_{1.7}Al_{0.3}(PO_4)_3$ (LATP). Error bound of calculated σ was estimated from the linear fitting of the Arrhenius plot (Supplementary Fig. 1).

Composition	E_a (eV)	σ at 300 K (mS cm^{-1})	Error bound [σ_{min}, σ_{max}] (mS cm^{-1})
$Li_{10}GeP_2S_{12}$ (AIMD)	0.23 ± 0.03	10	[3.40, 30.65]
$Li_{10}GeP_2S_{12}$ (Expt.) [1]	0.24	12	
cubic-$Li_7La_3Zr_2O_{12}$ (AIMD)	0.25 ± 0.02	1.3	[0.47, 3.49]
cubic-$Li_7La_3Zr_2O_{12}$ (Expt.)*[2–4]	0.31–0.34	0.31–0.51	
$Li_{1.3}Ti_{1.7}Al_{0.3}(PO_4)_3$ (AIMD)	0.25 ± 0.03	1.1	[0.30, 4.05]
$Li_{1.3}Ti_{1.7}Al_{0.3}(PO_4)_3$ (Expt.)**[5–7]	0.28–0.29	0.55–5	

* Ref. 4 are based on $Li_7La_3Zr_2O_{12}$ with 1.7 wt% Sr doping

** Ref. 6,7 are based on the $Li_{1.2}Ti_{1.8}Al_{0.2}(PO_4)_3$ composition

Supplementary Note 1. Statistical analysis of hopping events.

The concerted migration is also confirmed by the timing of hopping events in AIMD simulations (Supplementary Fig. 2). Each Li^+ hopping event is identified and counted at the time when a Li^+ changes its occupying site; the Li^+ spatial position is averaged over 2 ps to avoid counting local high-frequency vibrations. A plot of the total number of hopping events versus time shows that multiple ions hop within a short time interval (< 2 ps) in LGPS, LLZO and LATP (Supplementary Fig. 2a–c).

In addition, we performed a statistical analysis of the timing of all Li^+ hopping events in AIMD simulations. Li^+ hoppings that occurred within 1 ps were grouped as one individual concerted migration event, and the number of ions n in each migration event was determined. In LGPS, LLZO, and LATP, concerted migration ($n \geq 2$) dominates the overall diffusion (Supplementary Fig. 2a–c). This is further supported by the fact that in LGPS, the fast diffusion along the *c* direction illustrated in Fig. 3a is concerted migration, and most isolated hoppings (n=1) correspond to the Li^+ hopping in the *ab* direction. Thus, this statistical analysis of the timing of hopping events confirms concerted migration as the dominant diffusion mechanism in SICs.

Supplementary Note 2. Other concerted migration mechanisms in SICs.

The concerted migration mechanism in Fig. 3 is typical for LGPS, LLZO, and LATP, as observed in the AIMD simulations. Other concerted migrations observed in the AIMD simulations are also studied (Supplementary Fig. 3). In LGPS, the concerted migration of three Li ions along the *c* channel also shows a low activation energy barrier of 0.24 eV. In LATP, the same concerted migration mode as in Fig. 3c shows a slightly higher E_a, 0.32 eV, which is a result of different local Al/Ti configurations. In LLZO, the concerted migration modes are highly versatile. In all modes present in LLZO, multiple Li ions hop from their previous O and T sites into their nearest-neighbor T and O sites, respectively. These concerted migration processes are similar to the one illustrated in Fig. 3b except for the fact that different number of Li ions and different Li^+ sublattice configurations are involved. In addition, all LLZO migrations show low migration barriers (ranging from 0.18 to 0.29 eV). Therefore, while concerted migrations may show different modes in the disordered Li^+ sublattice, the energy barriers of those modes are similar to those of the typical concerted migrations shown in Fig. 3.

Supplementary Note 3. Diffusion model for concerted migration.

In the diffusion model in Fig. 4, the Coulomb interaction strength K among mobile ions was estimated from DFT calculations as:

$$K = \frac{E_{\mathrm{DFT}}(r_1) - E_{\mathrm{DFT}}(r_2)}{\frac{1}{r_1} - \frac{1}{r_2}}, \quad (1)$$

where $E_{\mathrm{DFT}}(r)$ is the DFT energy of the materials framework with two Li ions at a distance r. The distances r_1 and r_2 are chosen as the nearest and second-nearest neighboring Li sites, so that the energy difference between $E_{\mathrm{DFT}}(r_1)$ and $E_{\mathrm{DFT}}(r_2)$ is purely from the Coulomb interaction between these two Li ions. The Coulomb interaction strength K calculated in DFT is 2.7 eV·Å, 2.0 eV·Å, and 4.2 eV·Å for LGPS, LLZO, and LATP, respectively.

The arrangement of mobile ions in the diffusion model (Fig. 4) is similar to that of the Li^+ configuration in LATP (Fig. 3f). All ions were allowed to relax from the initial positions. After reaching the equilibrium, the high-energy sites remained occupied. The lowest energy migration pathway was calculated using the NEB method. The NEB calculation was converged to an energy tolerance of < 0.001 eV. We estimated the energy barrier of concerted migration in energy landscapes with different values of the barrier E_a and the unit lattice L (Supplementary Fig. 4). The barrier of concerted migration is significantly lower than the highest barrier of the energy landscape for all values of E_a and L tested.

In addition, we studied the diffusion model with different ion configurations, as shown in Supplementary Fig. 4c. In this model, the potential energy of the structural framework is the same as the energy landscape in Fig. 4a. Two ions (not plotted) are fixed at the position of −3.0 Å and 27.0 Å to provide the interactions from neighboring ions. Three high-energy sites are unoccupied, corresponding to an average site occupancy of ~ 67%, which is close to the average site occupancy in LGPS and LLZO. In this mobile-ion configuration, the concerted migration of two ions has a barrier of 0.2–0.4 eV (Supplementary Fig. 4d), for K ranging from 2–4 eV·Å, which is lower than the landscape barrier of 0.6 eV. As shown in the diffusion models with two different ion configurations, the concerted migration mechanism with low barriers is universal and can be activated with high-energy site occupancy, even though specific mobile ion configuration may vary for different orderings, compositions, and structural frameworks.

Supplementary Note 4. Features in non-SIC materials.

We studied Li_2S, $LiMn_2O_4$, and $LiTiS_2$ as representative materials of non-SICs. In these non-SICs, the probability density of Li ions at 900 K computed from AIMD simulations (Supplementary Fig. 5a, b) do not show the elongation features presented in the SICs (Fig. 2). In addition, $LiTiS_2$ has an energy landscape (Supplementary Fig. 5c) similar to that in Fig. 4b in contrast to the typical energy landscape of SIC materials (Fig. 4a).

Supplementary Note 5. Structural features and concerted migration in other ionic conductors.

We also verified the identified structural features and concerted migration mechanism in other Li ionic conductors, such as $Li_7P_3S_{11}$, $Li_{14}ZnGe_4O_{16}$ (LISICON), and β-Li_3PS_4. The activation energies of $Li_7P_3S_{11}$, $Li_{14}ZnGe_4O_{16}$, and β-Li_3PS_4 from AIMD simulations are 0.17 eV, 0.32 eV and 0.27 eV, respectively, which are in good agreement with experimental values at similar temperature ranges (Supplementary Fig. 11).[9–12] The energy landscape of single Li^+ migration (Supplementary Fig. 6–8) has high energy barriers: 0.39, 0.48, and 0.77 eV for $Li_7P_3S_{11}$, β-Li_3PS_4, and $Li_{14}ZnGe_4O_{16}$, respectively, and the high-energy sites are occupied. Thus, the concerted migration has lower migration barrier than the energy landscape. The distinctive part of the van Hove correlation function (Supplementary Fig. 6–8) also suggests concerted migration of Li ions. The Li^+ probability density in these materials (Supplementary Fig. 6–8) also shows elongation along the migration directions, similar to those in LGPS, LLZO, and LATP (Fig. 2d–f). Therefore, the key features of SICs are observed in these materials, and our proposed mechanism reduces the migration barrier in these SICs.

In $Li_{0.31}La_{0.56}TiO_3$ (LLTO), the activation energy computed in AIMD simulations is 0.26 eV, which is in good agreement with experimental values.[11,13] We also observed the concerted migration of multiple Li ions in the AIMD simulations. The migration barriers of such concerted migrations in NEB calculations are as low as 0.26–0.39 eV (Supplementary Fig. 9). The single-ion migration, which is also observed as a typical migration mode in AIMD simulations, has a similarly low energy barrier.[14] LLTO is slightly different from other SICs in that both single-ion and concerted migration contribute to ionic transport. Our proposed mechanism is still active and hence reduces the energy barrier of concerted migrations of multiple ions.

The identified mechanism and structural features are also observed in the Na super-ionic conductor $Na_3Zr_2Si_2PO_{12}$ (NASICON). The concerted migration of Na ions is observed and Na^+ probability densities show elongation along the diffusion path (Supplementary Fig. 10). The experimental activation energy of $Na_3Zr_2Si_2PO_{12}$ is 0.20–0.24 eV at temperatures greater than 450 K [15], but the energy landscape of single Na^+ to migrate within the structural framework has a higher energy barrier of 0.78 eV (Supplementary Fig. 7). In consistent with the mechanism in LATP, the occupied high-energy sites promote concerted migration with a reduced energy barrier.

For O ion conductors, we studied fluorite-structure Bi_2O_3, which has the same structure as other fast O ion conductors, such as CeO_2 and ZrO_2. In our calculations, the activation energy from AIMD simulations is 0.46 eV (Supplementary Fig. 12), which is in good agreement with experiments.[16] We found that concerted migration of two O^{2-} has the low energy barrier of 0.4 eV, similar to the barrier observed in the AIMD simulations. This barrier is lower than that for single ion migration, suggesting that our proposed mechanism is active in reducing the barrier for concerted migration.

Supplementary Note 6. Prediction and design of new Li ionic conductors.

We selected new structures $LiTaSiO_5$ (space group $P12_1/c1$, ICSD-39648) and $LiAlSiO_4$ (space group $P6_422$, ICSD-2929), as the model systems to demonstrate designing new fast ionic conductor materials. These compounds were selected by screening all compounds in the ICSD database with the following criteria: 1) No transition metal elements, such as V, Mn, Fe, Co and Ni, which are known to induce electronic conduction; 2) big bottleneck size > 0.5 Å of diffusion channel; and 3) Li percolation network with Li-Li site distance < 3 Å. Among many materials suggested by these criteria, we chose $LiTaSiO_5$ and $LiAlSiO_4$ as model systems, because they have not been studied for Li ion transport.

These compounds were doped through aliovalent cation substitution to introduce extra Li into high-energy Li sites. The Li sublattice of the doped structures was reordered to obtain a low-energy Li^+ configuration. $LiTaSiO_5$ shows relatively slow Li diffusion (E_a = 0.73±0.08 eV, σ_{300K}

$= 2.8\times10^{-7}$ mS cm^{-1}) with a high activation energy due to the high-barrier energy landscape (Supplementary Fig. 13). The doped $Li_{1.25}Ta_{0.75}Zr_{0.25}SiO_5$ is obtained by substituting Ta^{5+} with Zr^{4+}, and has extra Li ions occupying a fraction of high-energy B and C sites (Supplementary Fig. 13). As a result, concerted migration of Li ions is activated, leading to a significantly lower activation energy and higher ionic conductivity ($E_a = 0.23\pm0.01$ eV, $\sigma_{300K} = 4.3$ mS cm^{-1}).

Similarly, $Li_{1.25}Al_{1.25}Si_{0.75}O_4$, the doped $LiAlSiO_4$ structure resulting from the substitution of Si^{4+} with Al^{3+}, has additional Li ions occupying some high-energy B and C sites (Supplementary Fig. 14) in addition to the low-energy A and D sites originally occupied in $LiAlSiO_4$. Slow Li^+ diffusion in $LiAlSiO_4$ ($E_a = 0.43\pm0.05$ eV, $\sigma_{300K} = 0.01$ mS cm^{-1}) is significantly increased in $Li_{1.25}Al_{1.25}Si_{0.75}O_4$ ($E_a = 0.28\pm0.06$ eV, $\sigma_{300K} = 1.5$ mS cm^{-1}).

Supplementary References

1 Kamaya, N. *et al.* A lithium superionic conductor. *Nat. Mater.* **10**, 682–686 (2011).

2 Murugan, R., Thangadurai, V. & Weppner, W. Fast lithium ion conduction in garnet-type $Li_7La_3Zr_2O_{12}$. *Angew. Chem. Int. Ed.* **46**, 7778–7781 (2007).

3 Li, Y. *et al.* Ionic distribution and conductivity in lithium garnet $Li_7La_3Zr_2O_{12}$. *J. Power Sources* **209**, 278–281 (2012).

4 Dumon, A., Huang, M., Shen, Y. & Nan, C. W. High Li ion conductivity in strontium doped $Li_7La_3Zr_2O_{12}$ garnet. *Solid State Ionics* **243**, 36–41 (2013).

5 Aono, H., Sugimoto, E., Sadaoka, Y., Imanaka, N. & Adachi, G. Y. Ionic conductivity and sinterability of lithium titanium phosphate system. *Solid State Ionics* **40/41**, 38–42 (1990).

6 Arbi, K. *et al.* Lithium mobility in $Li_{1.2}Ti_{1.8}R_{0.2}(PO_4)_3$ compounds (R=Al, Ga, Sc, In) as followed by NMR and impedance spectroscopy. *Chem. Mater.* **16**, 255–262 (2004).

7 Arbi, K., Mandal, S., Rojo, J. & Sanz, J. Dependence of ionic conductivity on composition of fast ionic conductors $Li_{1+x}Ti_{2-x}Al_x(PO_4)_3$, $0\leq x\leq 0.7$. a parallel NMR and electric impedance study. *Chem. Mater.* **14**, 1091–1097 (2002).

8 He, X. & Mo, Y. Accelerated materials design of $Na_{0.5}Bi_{0.5}TiO_3$ oxygen ionic conductors based on first principles calculations. *Phys. Chem. Chem. Phys.* **17**, 18035–18044 (2015).

9 Seino, Y., Ota, T., Takada, K., Hayashi, A. & Tatsumisago, M. A sulphide lithium super ion conductor is superior to liquid ion conductors for use in rechargeable batteries. *Energy Environ. Sci.* **7**, 627–631 (2014).

10 Homma, K. *et al.* Crystal structure and phase transitions of the lithium ionic conductor Li_3PS_4. *Solid State Ionics* **182**, 53–58 (2011).

11 Knauth, P. Inorganic solid Li ion conductors: An overview. *Solid State Ionics* **180**, 911–916 (2009).

12 Bruce, P. & West, A. Ionic conductivity of LISICON solid solutions, $Li_{2+2x}Zn_{1-x}GeO_4$. *J. Solid State Chem.* **44**, 354–365 (1982).

13 Ma, C. *et al.* Mesoscopic framework enables facile ionic transport in solid electrolytes for Li batteries. *Adv. Energy Mater.* **6**, 1600053 (2016).

14 Catti, M. Short-range order and Li^+ ion diffusion mechanisms in $Li_5La_9\square_2(TiO_3)_{16}$ (LLTO). *Solid State Ionics* **183**, 1–6 (2011).

15 Baur, W., Dygas, J., Whitmore, D. & Faber, J. Neutron powder diffraction study and ionic conductivity of $Na_2Zr_2SiP_2O_{12}$ and $Na_3Zr_2Si_2PO_{12}$. *Solid State Ionics* **18**, 935–943 (1986).

16 Harwig, H. A. & Gerards, A. G. Electrical properties of the α, β, γ, and δ phases of bismuth sesquioxide. *J. Solid State Chem.* **26**, 265–274 (1978).
